# Chemical Composition of Myrtle (*Myrtus communis* L.) Berries Essential Oils as Observed in a Collection of Genotypes

**DOI:** 10.3390/molecules23102502

**Published:** 2018-09-29

**Authors:** Marianna Usai, Mauro Marchetti, Nicola Culeddu, Maurizio Mulas

**Affiliations:** 1 Department of Chemistry and Pharmacy, University of Sassari, Via Muroni 23/A, I-07100 Sassari, Italy; dsfusai@uniss.it; 2 Institute of Biomolecular Chemistry, National Research Council (CNR), Trav. La Crucca 3, 07100 Sassari, Italy; mauro@ss.cnr.it (M.M.); nicola.culeddu@icb.cnr.it (N.C.); 3 Department of Agriculture, University of Sassari, Via De Nicola 9, I-07100 Sassari, Italy

**Keywords:** *Myrtaceae* family, myrtle fruit, volatile composition, GC-MS, genetic variability

## Abstract

Myrtle (*Myrtus communis* L.) is a shrub spontaneously growing in the Mediterranean area. The leaf and fruit content of essential oils and phenolic compounds justify the wide use of the plant as medicinal and aromatic. Because of overexploitation of wild plants, a domestication process is in progress in different regions and the influence of the genotype variability on the chemical composition of fruit essential oils may be useful to breeding programs. Consequently, the analysis performed on a selected group of candidate clones growing in the same field collection in Sardinia is the object of this report. Forty-seven selections provided fully ripe fruits for essential oil extraction by hydrodistillation and Gas Chromatography-Mass Spectrometry (GC-MS) analysis. Only five candidate clones showed white fruits. The highest yield of essential oil was observed in the LAC31 genotype with 0.55 g·kg^−1^, while the samples BOS1, MON5, RUM4, RUM10, V4 and V8 showed values above 0.20 g·kg^−1^ and most of the genotypes under 0.10 g·kg^−1^. Geranyl acetate was the compound with the highest relative abundance. The second compound for relative abundance was the 1,8-cineole. Other compounds with high relative abundance were *α*-terpinyl acetate, methyleugenol, linalool, *α*-terpineol, *β*-caryophyllene, *α*-humulene, *Trans*-caryophyllene oxide, and humulene epoxide II.

## 1. Introduction

Myrtle (*Myrtus communis* L.) grows wild in the Mediterranean basin up to 800 m above sea level. Myrtle prospers in mild climates, fears frost but not drought, and prefers sandy, loose permeable soil with neutral or sub acid reaction. It is common in the Mediterranean maquis. In Sardinia and Corsica, it is a part of low Mediterranean maquis [1,2,3,4,5].

There are many scientific articles on the composition and biological activities of *Myrtus communis*; most studies on myrtle have focused on its volatile fraction. Due to its importance in the perfume and flavor industry, the chemical composition of myrtle essential oils was previously studied mainly in leaves from different geographic areas: Italy, Sardinia, Corsica, Tunisia, Algeria, Greece, Cyprus, Montenegro, Croatia, and Iran [6,7,8,9,10,11,12,13,14,15,16,17,18,19,20,21,22,23,24,25,26,27,28,29].

The presence of essential oils in all tissues is of fundamental importance to determine the antioxidant, antibiotic and antimutagenic properties of the myrtle biomass [21,22,24,30]. Several studies have indicated the activities of *Myrtus* chemical components [31,32,33]. Less information is available on myrtle essential oil from flowers [13,17,23,34].

In addition, less investigation was performed on the essential oils of berries and few papers have been published on these topics [23,34,35,36,37].

Boelens et al. [34] analyzed the hydrodistilled oils coming from Spanish wild-growing unripe and ripe fruits. Eighty components have been identified and quantified. The yields of the hydrodistilled oils were obtained for unripe and ripe fruits: 0.5% and 0.02% respectively. They found that during ripening the concentration of the main constituents changed: e.g., 1,8-cineole increased from 19.5% to 61.5% while myrtenyl acetate decreased from 33.0% to 0.1%. 

Jerkovic et al. [23] studied the Croatian myrtle fruit oils along the year and found that myrtenyl acetate (12.2–33.2%), 1,8-cineole + limonene (10.9–21.1%), *α*-pinene (4.0–15.3%), and linalool (4.7–7.7%) were the major constituents. Among them, linalool showed minimal quantitative changes. During the collecting period, high concentration of myrtenyl acetate was detected in September, while the concentrations of the four other quantitatively important compounds were highest in February, when the concentration of myrtenyl acetate was lowest. In this period, the fruit oil yields varied from 0.03 to 0.13%.

Pereira et al. [36] studied the composition of essential oil from Portuguese myrtle through the vegetative cycle. They found that Portuguese essential oils of myrtle berries are characterized by high content of limonene + 1,8-cineole (25.9%) and myrtenyl acetate (6.6%). *α*-pinene (9.7%) and linalool (36.5%) are also present at high level. These results indicate that Portuguese myrtle belongs to the group of myrtle genotypes, which characterized by the presence of myrtenyl acetate as one of the major components. 

Messaoud et al. [37] report of some Gas Chromatography-Mass Spectrometry (GC-MS) analyses of essential oils extracts from mature dark blue and white berries of Tunisian *Myrtus communis* samples growing at the same site, which allowed the identification of 33 chemical components. The oils from dark blue fruits showed high percentages of *α*-pinene (11.1%), linalool (11.6%), *α*-terpineol (15.7%), methyl eugenol (6.2%), and geraniol (3.7%). Myrtenyl acetate (20.3%) was found to be the major compound in the oils from white berries.

Brada et al. [38] studied the Algerian myrtle essential oil and the yield obtained from berries was 0.1%. Twenty-four constituents were identified, representing 89.5% of the berry oil analysis, the main components being: linalool (36.2%), followed by estragole (18.4%) and 1,8-cineole (11.4%). The oxygenated monoterpenes were the predominant chemical group (71.2%), followed by the sesquiterpenoids (16%). Monoterpenes (1.7%) and oxygenated sesquiterpenes (0.4%) were very low. Berry oil is characterized by a great amount of linalool, estragole, 1,8 cineole and an appreciable amount of bergamotene and *E-*caryophyllene.

Kafkas et al. [39] studied the volatile compounds of white and black myrtle from Turkey. Seven samples (identified by numbers) were collected in two different stations and fruit volatiles were extracted by HS/SPME. Thirty-one volatile compounds were identified in fruits. The lowest hexanal percentage was detected in type 2, while the highest was detected in type 4. Four ester compounds were detected in white myrtle types, whereas no ester was identified in black myrtle types. Linanyl-butyrate and linanyl-acetate were detected with higher percentages. Alcohols were detected as major compounds except type 16, whereas, terpenes compounds were detected as major compounds in type 16.

Among the detected terpene compounds, *α*-pinene was the major compound. Limonene was detected in white myrtle types, whereas, this compound was not detected in black types. Eucalyptol was detected in higher percentages in black myrtle (types 4 and 5, respectively) compared to the white types (2, 3, 8, 16 and 1, respectively). 

Mazza in 1983 [35] make the first GC-MS investigation on the volatile components of myrtle berries from Sardinian myrtle berries. He gave a detailed picture of the volatile components of myrtle berries analyzing the methanol extract of berries from Sardinia after centrifugation with water and extraction with pentane-methylene chloride that was used for GC-MS analyses. An alcoholic extract (60% ethanol) and commercial samples of liqueurs were also analyzed.

The extract obtained with solvent showed that α-pinene, limonene and 1,8-cineole represent 72% of the volatile fraction. Eleven hydrocarbons have been identified. Alcohols are 11% of volatile fraction and linalool and α-terpineol (being the most abundant) together reach 57% of total.

Tuberoso et al. [11] investigated the chemical composition of volatiles in Sardinian myrtle alcoholic extracts and essential oils. Although the content of monoterpenes represented 65.7–89.1% of the oil samples, some chemical constituents were remarkably different. For example, α-pinene ranged from 18.2% to 38.9%, δ-3-carene from 0.0 to 6.1%, *p*-cymene from 0.1% to 10.3%, limonene widely ranged from 3.7% to 44.5%, 1,8-cineole from 5.8% to 24.8%, γ-terpinene from 0.5% to 5.8%; terpinolene from 0.0% to 5.9%. Linalool widely ranged from 0.4% to 14.7%, terpenyl acetate from 0.1% to 5.4%, and geranyl acetate from 0.2% to 13.0%. The berries showed a moderate amount of sesquiterpenes representing 5.0% of the entire oil at the most.

Since ancient times, myrtle has been used as a medicinal plant. In Sardinia, it is very common the production of a myrtle liqueur [5]. Considering the high economic importance of myrtle industry in Sardinia the characterization of the genetic variability in wild and domesticated accessions may be a fundamental contribute to the breeding of the species [40,41,42]. 

Melito et al. in 2013 and in 2017 [40,41,42] studied genotypic variation and genetic diversity that were characterized using standard population genetics approaches. The level of genetic variability was high. The genetic data were compatible with the notion that myrtle has a mixed pollination system, including both out-pollination by insects and self-pollination. The candidate cultivars may represent an appropriate basis for directed breeding. All these selections are cultivated in Fenosu (Oristano) experimental field and represent a wide population usable to investigate chemical variations in these genotypes. In this field the production of different chemical profiles, in all parts of these plants, are regulated only by genetic differences because environmental condition is the same for all populations. In this view, considering the low level of information on the chemical composition of myrtle berries essential oils and the importance of these to determine the flavor of myrtle industry products, we investigated the chemical variation of berries essential oils with the aim to standardize the potential use of every clone selection.

## 2. Results and Discussion

**Figure 1:**
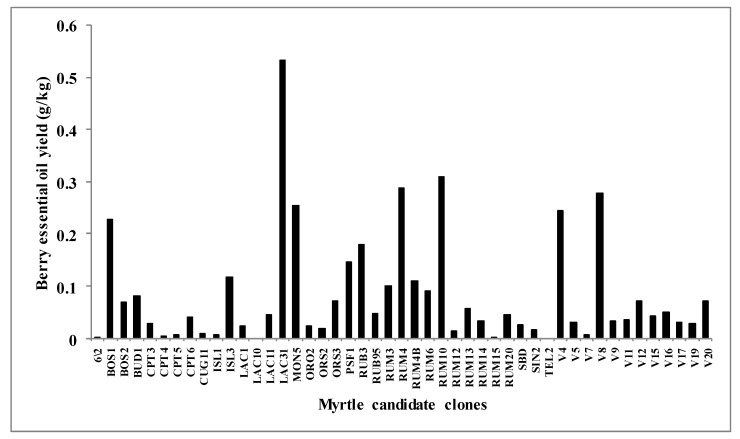
Yield of essential oils from 47 candidate clones of berries of *M. communis* L. The least significant difference value was 0.064 according to the application of Tukey’s test at *p* ≤ 0.05 level.

In our studies, we consider 47 different candidate clones: only five of *leucocarpa varietas* and the most part of *melanocarpa varietas*. The yield of essential oil of the berries was highly variable (Figure 1). Six candidate clones did not have enough quantity of fruits to guarantee a correct random harvesting and oil yield. LAC10 and TEL2, do not seem to produce essential oils in appreciable amount, while CPT5, RUM15, RUM13 and 6/2 gave yields around 0.005g·kg^−1^, but the investigation has not resulted in a GC chromatogram associable to typical components of essential oils but only to hydrocarbons probably attributable to waxes. The highest yield of essential oil was observed in the LAC31 genotype with 0.55 g·kg^−1^, while the samples BOS1, MON5, RUM4, RUM10, V4 and V8 showed values above 0.20 g·kg^−1^ and most of the genotypes under 0.10 g·kg^−1^.

GC-MS analysis of essential oils resulted in the detection of 92 compounds that are showed in the Table 1, Table 2, Table 3 and Table 4. The main components were: geranyl acetate for 13 genotypes; 1,8-cineole for 7 genotypes; *α*-terpinyl-acetate for 4 genotypes; linalool, *α*-humulene, *Trans*-caryophyllene oxide and *β*-caryophyllene for 3 genotypes; limonene for 2 genotypes; *α*-terpineol, bornyl acetate and humulene epoxide II respectively for 1 genotype each. *β*-caryophyllene was the compound present in all the genotypes and methyl eugenol in 40 selections. 

The compounds present only in one genotype with small quantities were the 2-methylbutanoic acid, 2 methylpropil ester, the *p*-mentha-1(7),8-diene, the linalyl acetate, the *β*-bisabolene, the ledol and the leptospermone iso.

Geranyl acetate was the compound with the highest relative abundance in all the population of candidate clones. It was present in 35 genotypes and the compound with the highest percentage in one essential oil, with the 50.95% in the candidate clone V5. The presence of geranyl acetate in the essential oils of the studied population gives a strong characterization to the same.

The second compound for relative abundance was the 1,8-cineole. It was present in 25 genotypes and the highest percentage was 43.26% in the candidate clone V4. Other compounds with high relative abundance were α-terpinyl acetate with the maximum of 23.56% in RUB95, methyleugenol with 19.66% in the sample LAC11, linalool with 35.10% in ISL3, *α*-terpineol with 23.21% in the RUM14 genotype, β-caryophyllene with 35.00% in MON5, *α*-humulene with 24.72% in LAC31, *Trans*-caryophyllene oxide with 25.69% in ORO2, and humulene epoxide II with 15.43% in ISL1.

The five genotypes with white berries showed limonene, 1,8-cineole, *α*-terpinyl acetate, and α-terpineol as main components but this chemotype was not exclusive of the candidate clones with white fruits.

The studied population of candidate clones had a high variability among genotypes and data on chemical composition of essential oils were quite different with respect to previously published data on samples from Sardinia and different other areas of Mediterranean region [25,34,35,36,37,38,39]. The recurrent association of main compounds was among α-terpinyl acetate, geranyl acetate, methyl eugenol and α-terpineol, as markedly evidenced in the candidate clones RUM6, BUB95, RUM20, CPT4, V9, V12, LAC1, ORS2, ORS3, SIN2, and PSF1 and only partially reported by other authors for Sardinia myrtle berry essential oils [11,35]. Other associations of main compounds were that of *α*-pinene, *p*-cymene, limonene, and 1,8-cineole, as reported for the selections RUB3, V8, RUM10, CPT6, V4, and BOS1; and that of limonene, 1,8-cineole, linalool, and geranyl acetate, characterizing RUM13, RUM14, and RUM4B. The association between linalool and geranyl acetate was also observed as characteristic of V17, V19, and V20. 

Many of the most important components of these possible chemotypes have been previously reported as part of myrtle berries essential oils [11,23,34,35,36,37,38,39]. However, some aspects appeared as new and not previously reported. Firstly, the relative scarcity of *α*-pinene and linalool, always indicated by previous studies as two of the most abundant compounds. Furthermore, the absence or low content of myrtenyl acetate is a result that differentiates our study from all the previous findings [11,23,34,35,36,37,38,39].

Finally, we detected both the presence of previously described chemotypes or associations of main components and the original nature of some chemical profiles not previously reported for myrtle berries. This was the case of RUM12 with prevalence of metyleugenol, trans-caryophyllene oxide and dihydroeugenyl pentanoate, a recently described compound for the myrtle leaf and flower essential oils [13], and 38 other compounds in a very complex essential oil. Other genotypes showed chemical profiles of the essential oils absolutely originals: CPT3 with neryl acetate, *α*-humulene and *α*-selinene as main components; V7 with *α*-humulene, *Trans*-caryophyllene oxide and humulene epoxide II; and ORO2 with neryl acetate, *Trans*-caryophyllene oxide, and selinene-11-en-4-*α*-ol.

**Figure 2:**
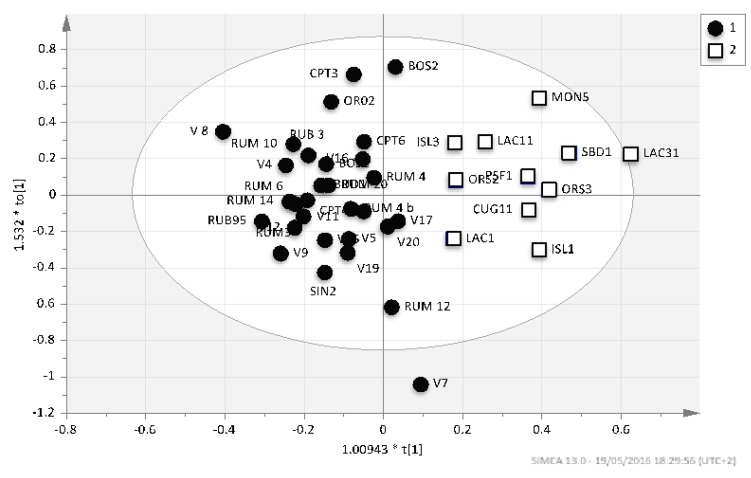
Genotypes distribution according to the two main components of variance as obtained by statistical multivariate analysis.

Application of multivariate analysis showed that the main components of the variance separate fairly genotypes in two groups based on essential oil chemical composition (Figure 2). We easily distinguish a chemotype that spreads in the South-East of the Sardinia and in all the localities above 300 m of altitude (white symbols) and another spreading all over the other localities where the genotypes were selected [41].

## 3. Experimental Section

### 3.1. Plant Materials and Essential Oils Distillation

The fruits have been harvested in the educational and experimental farm “Antonio Milella” located in San Quirico (Fenosu-Oristano, Central Wester Sardinia, Italy) in December 2015 when fully ripe. Among the 47 cultivars, only 5 are belonging to the variety *leucocarpa* DC, that means with white-yellow or withe-green fruits, the other 42 are belonging to the variety *melanocarpa* DC, that means with black-blue or purple fruits. The considered selections originate from different localities of Sardinia [5]. At least 15 plants represented every candidate clone. Mulas M. identified the analyzed plants. Voucher specimens have been deposited at the Herbarium SASSA (Sassari) of the Department of Chemistry and Pharmacy, University of Sassari under a collective number 514. 

To avoid a harvesting not representative we collected the fruits all at the same phenological stage (fully ripe) making sure to take plant material around all plants collecting material from the top, from the sides and from the base of threes. In the laboratory, the plant material was cleaned from other foreign parts (little branches, lives) and the samples were made as uniform as possible.

From every cultivar where collected about 2 kg of fruits and divided into three parts to replicate the analyses. After harvest, the clean fruits were kept in refrigerator at −20 °C until their extraction. Every sample of berries was chopped using a blender at low speed and the essential oil samples were obtained from the chopped berries by hydrodistillation for 4 h using a Clevenger-type apparatus. For every selection three extractions were performed. The extraction yields calculated as g·kg^−1^ of fresh material are reported in Figure 1. The oils were stored in sealed vials, at −20 °C, ready for the chemical analysis. 

### 3.2. Gas Chromatography-Mass Spectrometry (GC/MS) Analysis

GC: Three replicates of each sample were analyzed by using a Hewlett-Packard Model 5890A GC, equipped with a flame ionization detector and fitted with a 60 m × 0.25 mm (I.D.), thickness 0.25 μm ZB-5 fused silica capillary column (Phenomenex, Torrance CA, USA). Injection port and detector temperatures were maintained at 280 °C. 

The column temperature was programmed from 50 °C to 135 °C at 5 °C/min (1 min), 5 °C/min up 225°C (5 min), 5 °C/min up 260 °C and then held for 10 min. 

Samples of 0.2 μL (volume injection) were analyzed, diluted in hexane using 2,6-dimethylphenol as internal standard. Injection was performed using a split/splitless automatic injector HP 7673 and helium as carrier gas. Several measurements of peak areas were performed with a HP workstation with a threshold set to 0 and peak width to 0.02. The quantization of each compound was expressed as absolute weight percentage using internal standard and response factors (RFs). The detector RFs were determined for key components relative to 2,6-dimethylphenol and assigned to other components based on functional group and/or structural similarity, since oxygenated compounds have lower detectability by FID (Flame Ionization Detector) than hydrocarbons. The standards (Sigma-Aldrich, Fluka and Merck grade) were >95% also, and actual purity was checked by GC. Several response factor solutions were prepared that consisted of only four or five components (plus 2,6-dimethylphenol) to prevent interference from trace impurities. It is known that the oxygenated compounds have a lower sensitivity than the hydrocarbons to FID, we have calculated the response factor using a standard mixture of *α*-pinene, *α*-terpineol, neral, geranial, geranyl acetate and caryophyllene; in this mixture terpene accounted for 92% of the mixture, aldehydes 5% and alcohols, esters and sesquiterpenes 1% each. In our analyses we obtained that the RF of hydrocarbons was equal to 1 while for alcohols it was 0.80 and for esters 0.71. For this reason, we have multiplied the experimental data obtained for the following correction factors: hydrocarbons for 1, aldehydes and ketones for 1.24, alcohols for 1.28 and esters for 1.408.

GC/MS: MS analyses were carried out with an Agilent Technologies model 7820A connected with a MS detector 5977E MSD (Agilent), and using the same conditions and column described above. The column was connected to the ion source of the mass spectrometer. Mass units were monitored from 10 to 900 AMU at 70 eV. In the identification procedure we considered only the peaks from 40 to 900 AMU.

The identification of constituents was based on comparison of the *R*_t_ values and mass spectra with those obtained from authentic samples and/or the Nist and Wiley library spectra, or on the interpretation of the EI-fragmentation of the molecules [52,53].

### 3.3. Statistical Analysis

Oil yield data were processed for ANOVA by means of the software MSTAT-C and mean separation of was performed by application of the Tukey’s test at *p* ≤ 0.05 level of significance.

Data were submitted to multivariate statistical evaluation. Prior to chemometric analysis, setting the total integral areas to 100 normalized the data and the generated ASCII file was imported into Microsoft EXCEL for the addition of labels. The matrix was imported into SIMCA-P software version 12.0, (Umetrics AB, Umeå, Sweden) for statistical analysis.

## 4. Conclusions

The essential oil content of myrtle berries is quite low with respect to the yields that may be recovered by leaves or flowers of the same plant. Moreover, in six genotypes yields obtained with the hydrodistillation extraction system were insufficient for sample analysis. However, the importance of the essential oil composition for organoleptic properties of myrtle berries or of food and medicinal products obtained from their biomass is fundamental. 

Among the main constituents of the myrtle essential oils geranyl acetate is a compound with a floral or fruity rose aroma. Geranyl acetate is soluble in alcohol and is used as a flavoring ingredient where a sweet fruity or citrus aroma is desired. Many uses are also reported for 1,8-cineole as fragrance and flavoring agent in foods, candies, cough drops, and personal care products [54,55]. This compound is the chief constituent of the oil of eucalyptus and was also found in essential oils of laurel, rosemary, and many other plants. *α*-terpinyl acetate and *α*-terpineol have pleasant odor similar to lilac and are common ingredients in perfumes, cosmetics, and flavors [56]. Linalool is a fragrant monoterpene alcohol found in the essential oils of numerous aromatic plants. Linalool is largely used as fragrance component in perfumes, cosmetics, soaps, and detergents but also as flavoring agent in foods. Methyl eugenol is mainly used as fragrance ingredient in perfumes, toiletries, detergents, and flavor ingredient in baked goods. This substance is reasonably anticipated to be a human carcinogen [57]. *β*-caryophyllene and *Trans*-caryophyllene are natural bicyclic sesquiterpenes that are constituents of many essential oils. β-caryophyllene and *Trans*-caryophyllene are two of the chemical compounds that contributes to the spiciness of black pepper [58]. *α*-humulene and humulene epoxide II are components of the essential oil from the flowering cone of the hops plant (*Humulus lupulus*), from which derives they names. 

Most value of the myrtle products is on their fragrance and permanence of the aromatic compounds of the berries in the processed foods, such as the typical myrtle liqueurs [5,11]. The research carried out provides new information on the recurrence of some aromatic profiles in the genotypes selected from the wild populations growing in Sardinia, and the directions for the possible replication in the cultivation of the candidate clones showing the most appreciable chemical composition of the berry essential oil. Considering the increasing development of the myrtle as a new crop, in the next future will be possible to increase the quality value of yielded biomasses also by combining the aromatic profiles of the cultivated clones, to obtain the most appreciated or beneficial results.

## Figures and Tables

**Table 1:** Chemical composition of berry essential oil of ten (n. 1–10) myrtle genotypes.

			Genotype Number	1	2	3	4	5	6	7	8	9	10	ID ^z^	Ref.
			Selection	RUM6	RUM14	RUB3	RUB95	V8	RUM3	RUM4	RUM4B	RUM10	RUM12		
Rt	KI Apolar Lit.	KI apolar	Compound	%	%	%	%	%	%	%	%	%	%		
17.88	939	937	*α*-pinene	0.52 ± 0.02		11.88 ± 0.77		10.32 ± 0.26		2.90 ± 0.16	1.22 ± 0.02	8.54 ± 0.47		Std	
21.40	986	985	butanoic acid, 2-methyl-,2-methylpropyl ester									0.28 ± 0.03		MS-RI	[43]
21.6	1002	1001	*α*-phellandrene											Std	
21.93	1002	1002	*δ*-2-carene			1.56 ± 0.08		2.46 ± 0.41			0.57 ± 0.04	2.15 ± 0.09	0.05 ± 0.01	Std	
22.25	1005	1004	pseudolimonene											MS-RI	[44]
22.66	1025	1021	*p*-cymene	2.80 ± 0.11		4.44 ± 0.05	1.23 ± 0.06	9.70 ± 0.13	1.75 ± 0.12	4.86 ± 0.28	2.77 ± 0.19	8.66 ± 0.43	0.02 ± 0.01	Std	
22.90	1031	1029	limonene	3.04 ± 0.16	1.54 ± 0.08	7.34 ± 0.32	1.39 ± 0.08	26.80 ± 1.24	1.19 ± 0.09	4.12 ± 0.22	19.18 ± 0.97	20.91 ± 1.04		Std	
23.07	1035	1031	1,8-cineole	17.20 ± 0.47	5.34 ± 0.39	36.41 ± 0.76	9.38 ± 0.43	26.67 ± 1.37	11.50 ± 0.78	25.95 ± 0.89	8.49 ± 0.17	22.69 ± 1.29		Std	
24.49	1060	1057	*γ*-terpinene	0.64 ± 0.03		1.92 ± 0.10	0.39 ± 0.03	0.95 ± 0.05	0.80 ± 0.03	1.82 ± 0.03	1.21 ± 0.06	0.86 ± 0.05		Std	
26.08	1089	1063	*α*-terpinolene			0.94 ± 0.06					0.69 ± 0.02			Std	
26.55	1097	1094	linalool	2.32 ± 0.09	6.30 ± 0.46	2.03 ± 0.11	2.89 ± 0.15	6.39 ± 0.39	14.93 ± 0.86	13.23 ± 0.45	11.48 ± 1.04	7.60 ± 0.14		Std	
26.75	1112	1108	n-amyl isovalerate	3.24 ± 0.18	1.83 ± 0.03	3.26 ± 0.17	3.41 ± 0.16	3.64 ± 0.26	3.83 ± 0.12	4.24 ± 0.09	1.38 ± 0.05	3.79 ± 0.17		MS-RI	[45]
28.81	1113	1115	*Trans*-pinocarveol	0.89 ± 0.05		0.50 ± 0.02	0.83 ± 0.09					0.35 ± 0.03		MS-RI	
30.62	1130	1133	cosmene											MS-RI	[46]
30.63	1168	1161	*Trans*-p-mentha-1(7),8-dien-2-ol	0.53 ± 0.02	1.00 ± 0.04	0.30 ± 0.01	0.48 ± 0.03							MS-RI	
30.70	1177	1177	terpinen-4-ol	1.27 ± 0.06	1.87 ± 0.09	0.63 ± 0.03	1.09 ± 0.10		1.02 ± 0.07		1.23 ± 0.09	0.48 ± 0.06		Std	
30.98	1180	1180	*m*-cymen-8-ol											Std	
31.15	1183	1181	*p*-cymen-8-ol		0.67 ± 0.07							0.41 ± 0.05		Std	
31.32	1189	1190	*α*-terpineol	11.22 ± 0.51	23.21 ± 0.48	4.95 ± 0.54	9.76 ± 0.81	2.82 ± 0.18	11.02 ± 0.65	7.90 ± 0.18	5.29 ± 0.22	3.05 ± 0.18	0.72 ± 0.03	Std	
31.64	1192	1191	estragole	0.66 ± 0.03	0.85 ± 0.02	0.46 ± 0.06	0.97 ± 0.09		0.94 ± 0.09					MS-RI	
32.69	1217	1213	*Trans*-carveol										0.04 ± 0.01	MS-RI	
33.07	1230	1229	nerol											Std	
33.21	1245	1242	(2-*Z*)-3-hexenyl isovalerate											MS-RI	
34.01	1246	1248	carvone											MS-RI	
34.28	1253	1255	geraniol		1.22 ± 0.03								0.61 ± 0.03	Std	
34.32	1257	1256	linalyl acetate											Std	
34.67	1267	1257	geranial	1.12 ± 0.07		0.37 ± 0.04	0.83 ± 0.07		2.97 ± 0.12	5.45 ± 0.15	2.73 ± 0.13			Std	
35.92	1289	1290	bornyl acetate											Std	
36.37	1299	1312	carvacrol											Std	
34.25	1325	1322	methyl geraniate		1.38 ± 0.03			0.74 ± 0.09				0.77 ± 0.09	0.07 ± 0.01	Std	
37.45	1326	1326	6-isoprenyl-3-methoxymethoxy-3-methyl-ciclohexene (isomer) ^y^		0.63 ± 0.02			0.88 ± 0.15				0.51 ± 0.08	0.03 ± 0.01	MS	
37.60	1326	1326	6-isoprenyl-3-methoxymethoxy-3-methyl-ciclohexene (isomer) ^y^									0.98 ± 0.11		MS	
37.70	1326	1326	6-isoprenyl-3-methoxymethoxy-3-methyl-ciclohexene (isomer) ^y^											MS	
37.70	1332	1327	myrtenyl acetate	0.68 ± 0.04	1.40 ± 0.04		0.54 ± 0.02						0.05 ± 0.01	MS-RI	
38.14	1349	1351	*α*-terpinyl acetate	19.97 ± 0.32		8.57 ± 0.17	23.56 ± 1.25		11.15 ± 0.23	7.76 ± 0.20	3.92 ± 0.28		0.05 ± 0.01	MS-RI	
38.41	1362	1366	neryl acetate				0.41 ± 0.02		1.38 ± 0.06		0.50 ± 0.08		0.06 ± 0.01	Std	
38.79	1381	1379	geranyl acetate	12.30 ± 0.66	17.67 ± 0.59	5.46 ± 0.12	16.95 ± 1.03	0.99 ± 0.16	16.81 ± 1.01	9.61 ± 0.25	12.21 ± 0.94	1.00 ± 0.04	4.03 ± 0.08	Std	
39.60	1388	1388	*β*-cubebene								1.32 ± 0.11		0.01 ± 0.01	Std	
39.46	1391	1395	*β*-elemene				0.27 ± 0.02						0.07 ± 0.02	Std	
40.10	1404	1401	methyleugenol	4.52 ± 0.27	4.52 ± 0.12	1.93 ± 0.09	5.03 ± 0.13	0.66 ± 0.12	7.29 ± 0.45	3.00 ± 0.12	4.02 ± 0.32	0.73 ± 0.03	6.74 ± 0.12	MS-RI	
40.25			unknown 1									0.32 ± 0.03			
40.69	1428	1430	*β*-caryophyllene	1.87 ± 0.01	5.55 ± 0.15	0.79 ± 0.07	2.13 ± 0.06	0.57 ± 0.11	3.99 ± 0.29	5.12 ± 0.42	5.97 ± 0.41	0.66 ± 0.05	0.96 ± 0.04	Std	
40.69	1435	1439	*Trans*-α-bergamotene											MS-RI	
41.19	1437	1434	*γ*-elemene											Std	
41.47	1441	1443	aromadendrene										0.16 ± 0.01	Std	
41.82			unknown 2				0.33 ± 0.06								
42.21	1455	1456	*α*-humulene	2.37 ± 0.03	7.03 ± 0.18	1.27 ± 0.05	2.95 ± 0.17		1.71 ± 0.09	2.28 ± 0.10	1.83 ± 0.09	0.18 ± 0.01	1.92 ± 0.14	Std	
42.43	1457	1456	*(E)-β*-farnesene											MS-RI	
42.53	1457	1454	*α*-patchoulene											MS-RI	
43.04	1458	1458	phenethyl pivalate											MS-RI	
43.12			unknown 3										0.24 ± 0.01		
42.93	1460	1460	*(Z)*-methyl isoeugenol											MS-RI	
43.22	1460	1458	alloaromadendrene	1.81 ± 0.14	0.84 ± 0.06	0.62 ± 0.03	2.25 ± 0.12	0.71 ± 0.04				1.06 ± 0.05	1.87 ± 0.11	MS-RI	
43.33	1489	1490	*β*-selinene											Std	
43.51	1492	1492	*γ*-selinene											Std	
43.55	1498	1499	*α*-selinene	1.43 ± 0.07	0.59 ± 0.05	0.40 ± 0.03	1.94 ± 0.14	0.63 ± 0.03				0.86 ± 0.04	2.39 ± 0.15	Std	
43.61	1500	1502	bicyclogermacrene	0.46 ± 0.02	1.02 ± 0.08	0.39 ± 0.02	0.65 ± 0.03							Std	
43.68	1506	1512	*β*-bisabolene											MS-RI	
44.06	1521	1520	dihydroeugenyl butanoate						0.70 ± 0.03	0.25 ± 0.01	0.92 ± 0.08	0.11 ± 0.01	3.65 ± 0.20	MS-RI	[13]
44.23	1523	1520	*δ*-cadinene								1.43 ± 0.07		0.39 ± 0.02	Std	
44.70	1530	1532	zonarene								0.62 ± 0.03			MS-RI	
44.87	1546	1543	*α*-calacorene										0.33 ± 0.01	MS-RI	
44.92	1547	1546	selina-3,7(11)-diene											MS-RI	
45.12	1548	1548	*(Z)*-nerolidol								0.49 ± 0.03		0.70 ± 0.02	Std	
45.37	1550	1549	elemol								1.36 ± 0.07		0.41 ± 0.01	MS-RI	
45.41	1551	1553	ledol											MS-RI	
45.89	1578	1579	spathulenol	0.36 ± 0.02			0.51 ± 0.02				0.35 ± 0.01		2.77 ± 0.19	MS-RI	
45.97	1580	1581	*Cis*-caryophyllene oxide											MS-RI	
45.97	1583	1583	*Trans*-caryophyllene oxide	3.76 ± 0.03	5.63 ± 0.28	0.54 ± 0.04	2.72 ± 0.09	1.30 ± 0.07	3.03 ± 0.12	1.75 ± 0.03	4.75 ± 0.09	2.09 ± 0.13	14.73 ± 0.25	MS-RI	
46.30	1594	1595	*Cis*-arteannuic alcohol											MS-RI	[47]
46.31	1598	1596	carotol											MS-RI	[44]
46.32	1601	1600	guaiol		0.59 ± 0.05		0.34 ± 0.02						0.56 ± 0.02	Std	
46.18	1603	1603	*α*-dihydro (10,11)bi sabolol											MS-RI	
46.48	1606	1608	humulene epoxide II	2.43 ± 0.15	4.46 ± 0.18	0.73 ± 0.04	3.01 ± 0.15	0.32 ± 0.02	1.40 ± 0.07		0.82 ± 0.04	0.78 ± 0.03		MS-RI	[48]
46.80	1613	1617	isoleptospermone										2.36 ± 0.09	MS-RI	
46.82	1620	1623	leptospermone										3.78 ± 0.12	MS-RI	[49]
46.92			unknown 4	1.33 ± 0.08	2.14 ± 0.11	0.64 ± 0.03	1.78 ± 0.09	0.62 ± 0.04							
46.98	1631	1631	dihydroeugenyl pentanoate						2.15 ± 0.10	0.97 ± 0.06	1.75 ± 0.08	1.26 ± 0.07	6.79 ± 0.18	MS-RI	[13]
47.06			unknown 5									0.37 ± 0.01			
47.15	1632	1634	*γ*-eudesmol								0.63 ± 0.03		1.04 ± 0.05	Std	
47.23			Unknwon 6	0.65 ± 0.03	1.57 ± 0.07	0.21 ± 0.01	0.89 ± 0.04								
47.32	1641	1643	alloaromadendrene epoxide		0.58 ± 0.06		0.38 ± 0.03					0.53 ± 0.02	2.74 ± 0.18	MS-RI	
47.42			unknown 7												
47.43	1641	1641	5, *α* caryophylla-4(14),8(15)-dien-5-ol										2.47 ± 0.17	MS-RI	
47.55	1642	1642	epi-*α*-muurolol										0.51 ± 0.02	MS-RI	
47.83	1644	1644	*α*-selinen-3,11-en-6-ol	0.60 ± 0.04			0.71 ± 0.03				0.45 ± 0.02		0.55 ± 0.02	MS	
47.89	1660	1660	*α*-selinen-11-en-4-ol						0.42 ± 0.02			0.43 ± 0.03	3.23 ± 0.09	MS	
48.05	1663	1661	epi-globulol											MS-RI	
47.97	1675	1670	*β*-bisabolol										1.51 ± 0.05	Std	
48.34	1682	1682	ledene oxide II										0.46 ± 0.03	MS-RI	[50]
48.56	1700	1713	eudesm-7(11)-en-4-ol										0.29 ± 0.01	MS-RI	[51]
49.22			unknown 8									0.63 ± 0.04			
50.59	1725	1738	*α*-farnesol										0.20 ± 0.01	Std	
51.29	1972	1978	*n*-hexadecanoic acid											MS-RI	
54.25	2000	1999	eicosane											MS-RI	
			**Number of identified compounds**	26	24	25	29	18	21	17	30	29	40		

**^z^** ID = Identification methods. MS: by comparison of the Mass spectrum with those of the computer mass libraries Adams, Nist 11 and by interpretation of the mass spectra fragmentations. RI: by comparison of retention index with those reported in literature [8,37]. Std: by comparison of the retention time and mass spectrum of available authentic standards. MS: identification of Mass spectrum. No-polar column ZB-5. Data are the mean of three replicates ± standard deviation. ^y^ Tentatively identified.

**Table 2:** Chemical composition of berry essential oil of ten (n. 11–20) myrtle genotypes.

			Genotype Number	11	12	13	14	15	16	17	18	19	20	ID ^z^	Ref.
			Selection	RUM20	CPT3	CPT4	CPT6	V4	V5	V7	V9	V11	V12		
Rt	KI apolar Lit.	KI apolar	Compound	%	%	%	%	%	%	%	%	%	%		
17.88	939	937	*α*-pinene				3.18 ± 0.06	7.02 ± 0.36						Std	
21.40	986	985	butanoic acid, 2-methyl-,2-methylpropyl ester											MS-RI	[43]
21.6	1002	1001	*α*-phellandrene				0.33 ± 0.03							Std	
21.93	1002	1002	*δ*-2-carene				4.53 ± 0.38	1.19 ± 0.06						Std	
22.25	1005	1004	pseudolimonene				0.22 ± 0.02							MS-RI	[44]
22.66	1025	1021	*p*-cymene				12.83 ± 0.68	3.31 ± 0.04					1.04 ± 0.09	Std	
22.90	1031	1029	limonene				10.22 ± 0.55	5.99 ± 0.18					4.58 ± 0.28	Std	
23.07	1035	1031	1,8-cineole				18.72 ± 0.37	43.26 ± 1.96			0.13 ± 0.01		3.79 ± 0.19	Std	
24.49	1060	1057	*γ*-terpinene				11.37 ± 0.26	1.78 ± 0.08						Std	
26.08	1089	1063	*α*-terpinolene				5.69 ± 0.14	0.77 ± 0.05						Std	
26.55	1097	1094	linalool	1.01 ± 0.07		0.60 ± 0.03	9.94 ± 0.54	3.68 ± 0.17	1.61 ± 0.07		2.40 ± 0.19	2.77 ± 0.21	4.49 ± 0.21	Std	
26.75	1112	1108	n-amyl isovalerate				2.42 ± 0.08	3.07 ± 0.18			1.50 ± 0.12		2.07 ± 0.11	MS-RI	[45]
28.81	1113	1115	*Trans*-pinocarveol				0.25 ± 0.02				0.51 ± 0.03		0.42 ± 0.03	MS-RI	
30.62	1130	1133	cosmene									0.74 ± 0.09	0.47 ± 0.03	MS-RI	[46]
30.63	1168	1161	*Trans*-p-mentha-1(7),8-dien-2-ol				0.87 ± 0.11							MS-RI	
30.70	1177	1177	terpinen-4-ol	0.69 ± 0.03		0.42 ± 0.02	2.46 ± 0.15	0.69 ± 0.09				1.38 ± 0.07	1.50 ± 0.04	Std	
30.98	1180	1180	*m*-cymen-8-ol											Std	
31.15	1183	1181	*p*-cymen-8-ol				0.72 ± 0.06				0.57 ± 0.03	10.16 ± 0.51	0.39 ± 0.03	Std	
31.32	1189	1190	*α*-terpineol	8.23 ± 0.43	1.81 ± 0.09	4.90 ± 0.24	6.12 ± 0.23	6.82 ± 0.87			9.15 ± 0.46		11.51 ± 0.69	Std	
31.64	1192	1191	estragole								0.77 ± 0.04	0.66 ± 0.03	0.66 ± 0.03	MS-RI	
32.69	1217	1213	*Trans*-carveol								0.42 ± 0.02		0.36 ± 0.02	MS-RI	
33.07	1230	1229	nerol											Std	
33.21	1245	1242	(2-*Z*)-3-hexenyl isovalerate				0.34 ± 0.02				0.58 ± 0.03			MS-RI	
34.01	1246	1248	carvone											MS-RI	
34.28	1253	1255	geraniol				0.20 ± 0.01							Std	
34.32	1257	1256	linalyl acetate											Std	
34.67	1267	1257	geranial		0.64 ± 0.03	2.48 ± 0.18		1.34 ± 0.04	4.68 ± 0.22		0.77 ± 0.06	1.44 ± 0.07	1.82 ± 0.08	Std	
35.92	1289	1290	bornyl acetate											Std	
36.37	1299	1312	carvacrol											Std	
34.25	1325	1322	methyl geraniate	0.98 ± 0.06							0.45 ± 0.02	0.87 ± 0.06	0.50 ± 0.04	Std	
37.45	1326	1326	6-isoprenyl-3-methoxymethoxy-3-methyl-ciclohexene (isomer) ^y^								0.21 ± 0.01		0.41 ± 0.03	MS	
37.60	1326	1326	6-isoprenyl-3-methoxymethoxy-3-methyl-ciclohexene (isomer) ^y^	1.04 ± 0.08		0.57 ± 0.04			0.46 ± 0.02		0.63 ± 0.03		1.45 ± 0.09	MS	
37.70	1326	1326	6-isoprenyl-3-methoxymethoxy-3-methyl-ciclohexene (isomer) ^y^											MS	
37.70	1332	1327	myrtenyl acetate	1.11 ± 0.09		0.84 ± 0.05						0.81 ± 0.05	0.73 ± 0.05	MS-RI	
38.14	1349	1351	*α*-terpinyl acetate	12.43 ± 0.73	6.70 ± 0.13	20.24 ± 0.92		6.51 ± 0.39	14.4 ± 1.04		7.9 ± 0.43	18.56 ± 1.26	13.3 ± 0.93	MS-RI	
38.41	1362	1366	neryl acetate		16.54 ± 0.71	1.21 ± 0.05	4.14 ± 0.11		2.84 ± 0.16		0.47 ± 0.02	0.58 ± 0.04	0.78 ± 0.06	Std	
38.79	1381	1379	geranyl acetate	21.38 ± 1.66		43.27 ± 1.98		8.57 ± 0.46	50.95 ± 2.58		27.78 ± 2.01	16.79 ± 1.04	21.61 ± 1.82	Std	
39.60	1388	1388	*β*-cubebene											Std	
39.46	1391	1395	*β*-elemene		1.34 ± 0.07	1.15 ± 0.05								Std	
40.10	1404	1401	methyleugenol	12.32 ± 0.80	1.49 ± 0.08	12.16 ± 0.47	0.58 ± 0.02	2.68 ± 0.11	1.11 ± 0.05		12.34 ± 0.31	6.82 ± 0.38	7.28 ± 0.81	MS-RI	
40.25			unknown 1												
40.69	1428	1430	*β*-caryophyllene											Std	
40.69	1435	1439	*Trans*-α-bergamotene											MS-RI	
41.19	1437	1434	*γ*-elemene											Std	
41.47	1441	1443	aromadendrene		1.37 ± 0.09						0.16 ± 0.01			Std	
41.82			unknown 2											Std	
42.21	1455	1456	*α*-humulene	11.98 ± 0.78	18.92 ± 1.09	0.93 ± 0.02	0.72 ± 0.03	1.6 ± 0.08	2.48 ± 0.12	23.75 ± 1.87	3.11 ± 0.54	6.82 ± 0.41	3.14 ± 0.65	Std	
42.43	1457	1456	*(E)-β*-farnesene		0.99 ± 0.04									MS-RI	
42.53	1457	1454	*α*-patchoulene		0.71 ± 0.03									MS-RI	
43.04	1458	1458	phenethyl pivalate			0.34 ± 0.01			1.03 ± 0.05					MS-RI	
43.12			unknown 3												
42.93	1460	1460	*(Z)*-methyl isoeugenol								0.15 ± 0.01			MS-RI	
43.22	1460	1458	alloaromadendrene		8.17 ± 0.11	1.46 ± 0.04					0.22 ± 0.01	0.62 ± 0.04		MS-RI	
43.33	1489	1490	*β*-selinene											Std	
43.51	1492	1492	*γ*-selinene											Std	
43.55	1498	1499	*α*-selinene		8.94 ± 0.12	1.36 ± 0.03					0.13 ± 0.01			Std	
43.61	1500	1502	bicyclogermacrene											Std	
43.68	1506	1512	*β*-bisabolene											MS-RI	
44.06	1521	1520	dihydroeugenyl butanoate	1.33 ± 0.06	0.10 ± 0.01		0.30 ± 0.02		0.43 ± 0.03	7.82 ± 0.65	2.52 ± 0.15	0.85 ± 0.05	0.93 ± 0.08	MS-RI	[13]
44.23	1523	1520	*δ*-cadinene											Std	
44.70	1530	1532	zonarene				0.15 ± 0.01				0.18 ± 0.01			MS-RI	
44.87	1546	1543	*α*-calacorene											MS-RI	
44.92	1547	1546	selina-3,7(11)-diene											MS-RI	
45.12	1548	1548	*(Z)*-nerolidol											Std	
45.37	1550	1549	elemol	0.66 ± 0.04			0.28 ± 0.01							MS-RI	
45.41	1551	1553	ledol											MS-RI	
45.89	1578	1579	spathulenol		1.05 ± 0.05	1.94 ± 0.05					0.26 ± 0.01			MS-RI	
45.97	1580	1581	*Cis*-caryophyllene oxide											MS-RI	
45.97	1583	1583	*Trans*-caryophyllene oxide	5.81 ± 0.38	6.39 ± 0.35	1.95 ± 0.04	1.10 ± 0.04		8.65 ± 0.60	17.27 ± 0.91	5.52 ± 0.12	9.13 ± 0.26	3.94 ± 0.03	MS-RI	
46.30	1594	1595	*Cis*-arteannuic alcohol	0.70 ± 0.05										MS-RI	[47]
46.31	1598	1596	carotol						0.55 ± 0.04					MS-RI	[44]
46.32	1601	1600	guaiol		0.34 ± 0.02									Std	
46.18	1603	1603	*α*-dihydro (10,11)bi sabolol						0.44 ± 0.06		0.34 ± 0.02	1.09 ± 0.09	0.35 ± 0.02	MS-RI	
46.48	1606	1608	humulene epoxide II	5.80 ± 0.39	5.59 ± 0.42	0.63 ± 0.03	0.41 ± 0.02	0.55 ± 0.02	2.25 ± 0.08	13.68 ± 0.83	2.07 ± 0.04	9.62 ± 0.75	3.30 ± 0.47	MS-RI	[48]
46.80	1613	1617	isoleptospermone											MS-RI	
46.82	1620	1623	leptospermone			0.41 ± 0.02								MS-RI	[49]
46.92			unknown 4												
46.98	1631	1631	dihydroeugenyl pentanoate	2.70 ± 0.27		0.10 ± 0.01	0.15 ± 0.01	0.40 ± 0.02	1.07 ± 0.04	10.84 ± 0.58	2.52 ± 0.18	2.73 ± 0.19	3.46 ± 0.58	MS-RI	[13]
47.06			unknown 5												
47.15	1632	1634	*γ*-eudesmol								0.35 ± 0.02			Std	
47.23			Unknwon 6												
47.32	1641	1643	alloaromadendrene epoxide	1.54 ± 0.16	1.13 ± 0.06				0.47 ± 0.02	10.79 ± 0.49	0.75 ± 0.03	1.72 ± 0.12	0.81 ± 0.05	MS-RI	
47.42			unknown 7												
47.43	1641	1641	5, *α* caryophylla-4(14),8(15)-dien-5-ol	0.78 ± 0.05	0.38 ± 0.02				0.50 ± 0.02		0.65 ± 0.03	0.56 ± 0.04	0.41 ± 0.02	MS-RI	
47.55	1642	1642	epi-*α*-muurolol											MS-RI	
47.83	1644	1644	*α*-selinen-3,11-en-6-ol	0.52 ± 0.03	0.49 ± 0.03						0.74 ± 0.06			MS	
47.89	1660	1660	*α*-selinen-11-en-4 -ol		1.85 ± 0.11	1.75 ± 0.05					0.46 ± 0.02			MS	
48.05	1663	1661	epi-globulol											MS-RI	
47.97	1675	1670	*β*-bisabolol								0.21 ± 0.01			Std	
48.34	1682	1682	ledene oxide II											MS-RI	[50]
48.56	1700	1713	eudesm-7(11)-en-4-ol											MS-RI	[51]
49.22			unknown 8												
50.59	1725	1738	*α*-farnesol											Std	
51.29	1972	1978	*n*-hexadecanoic acid											MS-RI	
54.25	2000	1999	eicosane											MS-RI	
			Number of identified compounds	19	21	21	27	18	17	6	35	21	29		

**^z^** ID = Identification methods. MS: by comparison of the Mass spectrum with those of the computer mass libraries Adams, Nist 11 and by interpretation of the mass spectra fragmentations. RI: by comparison of retention index with those reported in literature [8,37]. Std: by comparison of the retention time and mass spectrum of available authentic standards. MS: identification of Mass spectrum. No-polar column ZB-5. Data are the mean of three replicates ± standard deviation. ^y^ Tentatively identified.

**Table 3:** Chemical composition of berry essential oil of ten (n. 21–30) myrtle genotypes.

			Genotype Number	21	22	23	24	25	26	27	28	29	30	ID ^z^	Ref.
			Selection	V15	V16	V17	V19	V20	LAC1	LAC11	LAC31	BOS1	BOS2		
Rt	KI Apolar Lit.	KI Apolar	Compound	%	%	%	%	%	%	%	%	%	%		
17.88	939	937	*α*-pinene								0.35 ± 0.02	5.59 ± 0.21		Std	
21.40	986	985	butanoic acid, 2-methyl-,2-methylpropyl ester											MS-RI	[43]
21.6	1002	1001	*α*-phellandrene				0.23 ± 0.02		0.19 ± 0.01					Std	
21.93	1002	1002	*δ*-2-carene											Std	
22.25	1005	1004	pseudolimonene											MS-RI	[44]
22.66	1025	1021	*p*-cymene		2.91 ± 0.06			0.94 ± 0.05			0.66 ± 0.04	2.74 ± 0.13		Std	
22.90	1031	1029	limonene		12.87 ± 0.98	1.21 ± 0.04		1.19 ± 0.07			0.38 ± 0.02	9.08 ± 0.45		Std	
23.07	1035	1031	1,8-cineole		14.01 ± 0.69	2.46 ± 0.09		2.22 ± 0.13	0.09 ± 0.01		2.65 ± 0.14	41.18 ± 2.10		Std	
24.49	1060	1057	*γ*-terpinene		2.70 ± 0.10			0.59 ± 0.04		5.27 ± 0.41	0.39 ± 0.03	1.97 ± 0.09		Std	
26.08	1089	1063	*α*-terpinolene		0.95 ± 0.07			0.24 ± 0.02			0.53 ± 0.04	1.00 ± 0.06		Std	
26.55	1097	1094	linalool	2.70 ± 0.05	7.83 ± 0.19	28.71 ± 1.24	10.86 ± 0.59	34.04 ± 2.16	1.75 ± 0.01		10.63 ± 0.61	3.46 ± 0.14	1.90 ± 0.09	Std	
26.75	1112	1108	n-amyl isovalerate	0.33 ± 0.02	3.18 ± 0.15	2.18 ± 0.09	0.50 ± 0.04	1.92 ± 0.16	0.09 ± 0.01					MS-RI	[45]
28.81	1113	1115	*Trans*-pinocarveol	0.36 ± 0.02				0.35 ± 0.04	0.17 ± 0.01					MS-RI	
30.62	1130	1133	cosmene		1.29 ± 0.07	0.94 ± 0.04	0.43 ± 0.03	0.61 ± 0.07	0.60 ± 0.05	0.93 ± 0.06				MS-RI	[46]
30.63	1168	1161	*Trans*-p-mentha-1(7),8-dien-2-ol											MS-RI	
30.70	1177	1177	terpinen-4-ol	0.74 ± 0.04	2.00 ± 0.08	1.74 ± 0.07	0.55 ± 0.04	0.90 ± 0.06	0.84 ± 0.06	2.63 ± 0.10	1.56 ± 0.07			Std	
30.98	1180	1180	*m*-cymen-8-ol											Std	
31.15	1183	1181	*p*-cymen-8-ol		0.60 ± 0.03		0.13 ± 0.01		0.58 ± 0.04	0.47 ± 0.03				Std	
31.32	1189	1190	*α*-terpineol	5.14 ± 0.12	12.50 ± 0.24	11.87 ± 0.86	2.65 ± 0.10	5.80 ± 0.27	3.68 ± 0.17	17.55 ± 0.84	5.73 ± 0.26	3.14 ± 0.19	4.68 ± 0.20	Std	
31.64	1192	1191	estragole	0.30 ± 0.02	0.75 ± 0.04	0.77 ± 0.06	0.43 ± 0.03	0.35 ± 0.04	0.29 ± 0.02	0.65 ± 0.04				MS-RI	
32.69	1217	1213	*Trans*-carveol						0.09 ± 0.01					MS-RI	
33.07	1230	1229	nerol				0.16 ± 0.01				0.17 ± 0.01			Std	
33.21	1245	1242	(2-*Z*)-3-hexenyl isovalerate	0.25 ± 0.01			0.22 ± 0.01		0.15 ± 0.01					MS-RI	
34.01	1246	1248	carvone											MS-RI	
34.28	1253	1255	geraniol	0.94 ± 0.06			0.52 ± 0.03							Std	
34.32	1257	1256	linalyl acetate				4.94 ± 0.05							Std	
34.67	1267	1257	geranial			2.72 ± 0.09		0.55 ± 0.04	1.36 ± 0.07	1.75 ± 0.06	7.63 ± 0.39	1.16 ± 0.06	1.58 ± 0.12	Std	
35.92	1289	1290	bornyl acetate				0.12 ± 0.01		0.12 ± 0.01	24.29 ± 1.16				Std	
36.37	1299	1312	carvacrol				0.10 ± 0.01		0.12 ± 0.01					Std	
34.25	1325	1322	methyl geraniate	1.01 ± 0.07			0.30 ± 0.02	0.49 ± 0.03						Std	
37.45	1326	1326	6-isoprenyl-3-methoxymethoxy-3-methyl-ciclohexene (isomer) ^y^	0.41 ± 0.03			0.28 ± 0.02							MS	
37.60	1326	1326	6-isoprenyl-3-methoxymethoxy-3-methyl-ciclohexene (isomer) ^y^	0.80 ± 0.05	1.06 ± 0.07		0.65 ± 0.05				0.22 ± 0.01			MS	
37.70	1326	1326	6-isoprenyl-3-methoxymethoxy-3-methyl-ciclohexene (isomer) ^y^	1.08 ± 0.07	1.21 ± 0.06		0.33 ± 0.02		0.12 ± 0.01					MS	
37.70	1332	1327	myrtenyl acetate			0.46 ± 0.03	0.10 ± 0.01	0.42 ± 0.03			0.20 ± 0.01			MS-RI	
38.14	1349	1351	*α*-terpinyl acetate			7.36 ± 0.13	3.35 ± 0.16		8.45 ± 0.66		8.28 ± 0.45	4.21 ± 0.22	6.62 ± 0.33	MS-RI	
38.41	1362	1366	neryl acetate	0.31 ± 0.02		1.17 ± 0.06	1.36 ± 0.07		0.41 ± 0.03		0.90 ± 0.05		15.94 ± 0.78	Std	
38.79	1381	1379	geranyl acetate	12.56 ± 0.85	7.34 ± 0.18	17.93 ± 0.63	17.32 ± 0.96	6.88 ± 0.37	15.15 ± 1.03		10.23 ± 0.52	8.31 ± 0.41		Std	
39.60	1388	1388	*β*-cubebene					0.42 ± 0.03	0.37 ± 0.02					Std	
39.46	1391	1395	*β*-elemene	0.33 ± 0.02					0.46 ± 0.03	1.46 ± 0.07		1.54 ± 0.08	2.37 ± 0.11	Std	
40.10	1404	1401	methyleugenol	11.14 ± 0.78	9.88 ± 0.52	9.20 ± 0.19	6.41 ± 0.18	5.79 ± 0.26	10.03 ± 0.55	19.66 ± 1.02	3.35 ± 0.17	3.65 ± 0.18	9.17 ± 0.56	MS-RI	
40.25			unknown 1												
40.69	1428	1430	*β*-caryophyllene	6.76 ± 0.14	8.37 ± 0.09	4.47 ± 0.08	2.71 ± 0.11	5.25 ± 0.29	0.25 ± 0.02	4.24 ± 0.23	11.67 ± 0.56	5.31 ± 0.25	23.02 ± 1.14	Std	
40.69	1435	1439	*Trans*-α-bergamotene				0.17 ± 0.02				0.24 ± 0.02			MS-RI	
41.19	1437	1434	*γ*-elemene	0.27 ± 0.02										Std	
41.47	1441	1443	aromadendrene						0.17 ± 0.01	0.61 ± 0.05			1.70 ± 0.09	Std	
41.82			unknown 2											Std	
42.21	1455	1456	*α*-humulene	1.92 ± 0.13	2.23 ± 0.17	1.93 ± 0.05	6.27 ± 0.15	9.42 ± 0.48	0.28 ± 0.01	2.23 ± 0.11	24.72 ± 1.65	1.39 ± 0.06	4.40 ± 0.24	Std	
42.43	1457	1456	*(E)-β*-farnesene											MS-RI	
42.53	1457	1454	*α*-patchoulene						0.13 ± 0.01					MS-RI	
43.04	1458	1458	phenethyl pivalate	2.56 ± 0.16	1.18 ± 0.06		0.17 ± 0.01	0.55 ± 0.04	0.13 ± 0.01					MS-RI	
43.12			unknown 3												
42.93	1460	1460	*(Z)*-methyl isoeugenol											MS-RI	
43.22	1460	1458	alloaromadendrene				0.19 ± 0.01		4.95 ± 0.24			2.60 ± 0.16	7.99 ± 0.49	MS-RI	
43.33	1489	1490	*β*-selinene							3.22 ± 0.16				Std	
43.51	1492	1492	*γ*-selinene							3.31 ± 0.15				Std	
43.55	1498	1499	*α*-selinene				0.30 ± 0.02		3.06 ± 0.19					Std	
43.61	1500	1502	bicyclogermacrene				1.45 ± 0.06			1.08 ± 0.05				Std	
43.68	1506	1512	*β*-bisabolene											MS-RI	
44.06	1521	1520	dihydroeugenyl butanoate	1.39 ± 0.63	0.74 ± 0.08	1.07 ± 0.04	0.88 ± 0.04	1.62 ± 0.07	1.37 ± 0.05	0.10 ± 0.01	0.25 ± 0.02			MS-RI	[13]
44.23	1523	1520	*δ*-cadinene						0.14 ± 0.01					Std	
44.70	1530	1532	zonarene	1.28 ± 0.71			1.04 ± 0.05	0.30 ± 0.02	0.28 ± 0.02	0.43 ± 0.03	0.75 ± 0.05			MS-RI	
44.87	1546	1543	*α*-calacorene						0.15 ± 0.01					MS-RI	
44.92	1547	1546	selina-3,7(11)-diene				0.31 ± 0.02				0.92 ± 0.03			MS-RI	
45.12	1548	1548	*(Z)*-nerolidol				0.41 ± 0.03			0.33 ± 0.02				Std	
45.37	1550	1549	elemol				0.28 ± 0.02		0.09 ± 0.01		2.29 ± 0.12		1.84 ± 0.13	MS-RI	
45.41	1551	1553	ledol											MS-RI	
45.89	1578	1579	spathulenol				0.16 ± 0.01		2.38 ± 0.13	1.53 ± 0.06				MS-RI	
45.97	1580	1581	*Cis*-caryophyllene oxide											MS-RI	
45.97	1583	1583	*Trans*-caryophyllene oxide	15.68 ± 0.98	3.83 ± 0.14	2.07 ± 0.06	4.37 ± 0.14	5.72 ± 0.22	10.18 ± 0.64	2.13 ± 0.09	1.29 ± 0.10		0.97 ± 0.07	MS-RI	
46.30	1594	1595	*Cis*-arteannuic alcohol										2.88 ± 0.16	MS-RI	[47]
46.31	1598	1596	carotol											MS-RI	[44]
46.32	1601	1600	guaiol											Std	
46.18	1603	1603	*α*-dihydro (10,11)bi sabolol	0.35 ± 0.02			0.87 ± 0.09		0.49 ± 0.04		0.34 ± 0.02			MS-RI	
46.48	1606	1608	humulene epoxide II	2.86 ± 0.15	0.87 ± 0.04	0.83 ± 0.07	7.26 ± 0.68	6.01 ± 0.31	4.88 ± 0.25	0.62 ± 0.04	2.54 ± 0.11			MS-RI	[48]
46.80	1613	1617	isoleptospermone											MS-RI	
46.82	1620	1623	leptospermone						1.17 ± 0.07					MS-RI	[49]
46.92			unknown 4							0.36 ± 0.02					
46.98	1631	1631	dihydroeugenyl pentanoate	4.49 ± 0.36	1.06 ± 0.06	0.89 ± 0.05	2.21 ± 0.14	2.51 ± 0.09						MS-RI	[13]
47.06			unknown 5						1.62 ± 0.10						
47.15	1632	1634	*γ*-eudesmol											Std	
47.23			Unknwon 6					0.66 ± 0.04	0.58 ± 0.03						
47.32	1641	1643	alloaromadendrene epoxide	1.72 ± 0.12			2.01 ± 0.16	1.36 ± 0.08	0.86 ± 0.04		0.56 ± 0.04			MS-RI	
47.42			unknown 7												
47.43	1641	1641	5, *α* caryophylla-4(14),8(15)-dien-5-ol	1.55 ± 0.11			0.52 ± 0.04	0.55 ± 0.04	0.96 ± 0.04					MS-RI	
47.55	1642	1642	epi-*α*-muurolol											MS-RI	
47.83	1644	1644	*α*-selinen-3,11-en-6-ol	0.23 ± 0.01					0.27 ± 0.02	2.51 ± 0.13	0.92 ± 0.05			MS	
47.89	1660	1660	*α*-selinen-11-en-4 -ol	0.64 ± 0.05			1.59 ± 0.07	0.31 ± 0.02	4.14 ± 0.21					MS	
48.05	1663	1661	epi-globulol						0.39 ± 0.02				3.16 ± 0.19	MS-RI	
47.97	1675	1670	*β*-bisabolol	0.78 ± 0.05				0.24 ± 0.02	0.89 ± 0.03					Std	
48.34	1682	1682	ledene oxide II						0.12 ± 0.01					MS-RI	[50]
48.56	1700	1713	eudesm-7(11)-en-4-ol						0.06 ± 0.01		0.29 ± 0.02			MS-RI	[51]
49.22			unknown 8												
50.59	1725	1738	*α*-farnesol				0.29 ± 0.03		0.21 ± 0.02					Std	
51.29	1972	1978	*n*-hexadecanoic acid	0.35 ± 0.02					0.14 ± 0.01					MS-RI	
54.25	2000	1999	eicosane	0.66 ± 0.04			0.95 ± 0.06		0.24 ± 0.02					MS-RI	
			Number of identified compounds	32	23	20	45	31	52	24	30	16	15		

**^z^** ID = Identification methods. MS: by comparison of the Mass spectrum with those of the computer mass libraries Adams, Nist 11 and by interpretation of the mass spectra fragmentations. RI: by comparison of retention index with those reported in literature [8,37]. Std: by comparison of the retention time and mass spectrum of available authentic standards. MS: identification of Mass spectrum. No-polar column ZB-5. Data are the mean of three replicates ± standard deviation. ^y^ Tentatively identified.

**Table 4:** Chemical composition of berry essential oil of eleven (n. 31–41) myrtle genotypes.

			Genotype Number	31	32	33	34	35	36	37	38	39	40	41	ID ^z^	Ref.
			Selection	ORS2	ORS3	ISL3	BUD1	CUG11	ORO2	ISL1	SBD	SIN2	MON5	PSF1		
Rt	KI apolar Lit.	KI apolar	Compound													
17.88	939	937	*α*-pinene											0.36 ± 0.02	Std	
21.40	986	985	butanoic acid, 2-methyl-,2-methylpropyl ester												MS-RI	[43]
21.6	1002	1001	*α*-phellandrene												Std	
21.93	1002	1002	*δ*-2-carene												Std	
22.25	1005	1004	pseudolimonene												MS-RI	[44]
22.66	1025	1021	*p*-cymene		0.22 ± 0.01		0.58 ± 0.04				0.77 ± 0.05			1.03 ± 0.06	Std	
22.90	1031	1029	limonene				0.64 ± 0.05				0.62 ± 0.04			0.69 ± 0.05	Std	
23.07	1035	1031	1,8-cineole		0.57 ± 0.04	6.79 ± 0.37	2.25 ± 0.12				3.12 ± 0.22	0.55 ± 0.03		7.75 ± 0.30	Std	
24.49	1060	1057	*γ*-terpinene				0.35 ± 0.02				1.41 ± 0.11			0.64 ± 0.05	Std	
26.08	1089	1063	*α*-terpinolene				0.56 ± 0.04				1.72 ± 0.12			0.92 ± 0.06	Std	
26.55	1097	1094	linalool		3.41 ± 0.17	35.10 ± 2.36	2.02 ± 0.10				13.61 ± 0.89	10.02 ± 0.51	1.43 ± 0.06	2.39 ± 0.11	Std	
26.75	1112	1108	n-amyl isovalerate									0.16 ± 0.01	3.10 ± 0.16	0.23 ± 0.01	MS-RI	[45]
28.81	1113	1115	*Trans*-pinocarveol		0.17 ± 0.01							0.58 ± 0.03			MS-RI	
30.62	1130	1133	cosmene												MS-RI	[46]
30.63	1168	1161	*Trans*-p-mentha-1(7),8-dien-2-ol		5.80 ± 0.32										MS-RI	
30.70	1177	1177	terpinen-4-ol		3.79 ± 0.13		1.30 ± 0.06				3.19 ± 0.19	4.14 ± 0.28		3.42 ± 0.19	Std	
30.98	1180	1180	*m*-cymen-8-ol									0.28 ± 0.02		0.38 ± 0.02	Std	
31.15	1183	1181	*p*-cymen-8-ol		0.95 ± 0.06							0.30 ± 0.02		0.28 ± 0.02	Std	
31.32	1189	1190	*α*-terpineol	2.93 ± 0.18	6.90 ± 0.34	5.10 ± 0.24	3.94 ± 0.18	1.00 ± 0.06		0.12 ± 0.01	13.93 ± 1.03	8.53 ± 0.42	2.78 ± 0.14	10.04 ± 0.55	Std	
31.64	1192	1191	estragole		0.28 ± 0.01							0.59 ± 0.03		0.45 ± 0.03	MS-RI	
32.69	1217	1213	*Trans*-carveol									0.10 ± 0.01			MS-RI	
33.07	1230	1229	nerol									0.27 ± 0.02			Std	
33.21	1245	1242	(2-*Z*)-3-hexenyl isovalerate									0.17 ± 0.01			MS-RI	
34.01	1246	1248	carvone												MS-RI	
34.28	1253	1255	geraniol	1.57 ± 0.08				0.16 ± 0.01		0.41 ± 0.03	1.29 ± 0.87	6.05 ± 0.34	1.27 ± 0.10		Std	
34.32	1257	1256	linalyl acetate												Std	
34.67	1267	1257	geranial		1.80 ± 0.07		0.98 ± 0.05							2.32 ± 0.12	Std	
35.92	1289	1290	bornyl acetate		0.56 ± 0.03							0.13 ± 0.01			Std	
36.37	1299	1312	carvacrol		0.28 ± 0.02										Std	
34.25	1325	1322	methyl geraniate									0.09 ± 0.01		0.50 ± 0.04	Std	
37.45	1326	1326	6-isoprenyl-3-methoxymethoxy-3-methyl-ciclohexene (isomer) ^y^		0.19 ± 0.01							0.24 ± 0.02			MS	
37.60	1326	1326	6-isoprenyl-3-methoxymethoxy-3-methyl-ciclohexene (isomer) ^y^		0.25 ± 0.01							0.80 ± 0.04			MS	
37.70	1326	1326	6-isoprenyl-3-methoxymethoxy-3-methyl-ciclohexene (isomer) ^y^		0.24 ± 0.01		0.44 ± 0.03					0.27 ± 0.02			MS	
37.70	1332	1327	myrtenyl acetate		0.51 ± 0.04							0.69 ± 0.05			MS-RI	
38.14	1349	1351	*α*-terpinyl acetate	21.10 ± 0.96	12.68 ± 0.86		6.67 ± 0.36	3.90 ± 0.16		1.15 ± 0.06		17.70 ± 1.06	2.85 ± 0.13	20.25 ± 1.03	MS-RI	
38.41	1362	1366	neryl acetate	0.61 ± 0.04	0.60 ± 0.04		0.26 ± 0.02	0.18 ± 0.01	9.73 ± 0.81	0.57 ± 0.07		0.91 ± 0.06		0.40 ± 0.02	Std	
38.79	1381	1379	geranyl acetate	24.08 ± 1.23	23.84 ± 1.45	8.62 ± 0.46	10.46 ± 0.51	14.31 ± 0.79		9.46 ± 0.54	10.75 ± 0.62	27.53 ± 1.85	21.42 ± 1.05	20.09 ± 0.96	Std	
39.60	1388	1388	*β*-cubebene		0.24 ± 0.02		1.59 ± 0.07							0.02 ± 0.01	Std	
39.46	1391	1395	*β*-elemene	0.63 ± 0.05	0.29 ± 0.02		5.94 ± 0.30					1.37 ± 0.06			Std	
40.10	1404	1401	methyleugenol	16.49 ± 0.76	9.82 ± 0.56	2.94 ± 0.14	7.03 ± 0.41	8.34 ± 0.48	0.88 ± 0.06	6.37 ± 0.26	8.53 ± 0.46	6.72 ± 0.41	7.42 ± 0.36	7.97 ± 0.35	MS-RI	
40.25			unknown 1													
40.69	1428	1430	*β*-caryophyllene	8.36 ± 0.47	0.30 ± 0.02	8.81 ± 0.42	9.91 ± 0.58	6.12 ± 0.39	1.09 ± 0.07	0.14 ± 0.01	22.26 ± 1.85	1.44 ± 0.07	35.0 ± 2.04	7.44 ± 0.32	Std	
40.69	1435	1439	*Trans*-α-bergamotene		0.20 ± 0.01										MS-RI	
41.19	1437	1434	*γ*-elemene					0.16 ± 0.01				0.21 ± 0.02			Std	
41.47	1441	1443	aromadendrene	0.84 ± 0.06	0.61 ± 0.04		0.41 ± 0.03	0.20 ± 0.01				0.27 ± 0.02			Std	
41.82			unknown 2												Std	
42.21	1455	1456	*α*-humulene	1.64 ± 0.05	0.41 ± 0.03	26.37 ± 1.32	5.09 ± 0.26	4.14 ± 0.28		1.35 ± 0.06	7.80 ± 0.34	0.91 ± 0.05	5.09 ± 0.25	1.86 ± 0.09	Std	
42.43	1457	1456	*(E)-β*-farnesene				0.46 ± 0.03	0.12 ± 0.01	0.99 ± 0.06						MS-RI	
42.53	1457	1454	*α*-patchoulene	0.53 ± 0.04	0.46 ± 0.03		1.72 ± 0.06	0.15 ± 0.01				0.39 ± 0.03			MS-RI	
43.04	1458	1458	phenethyl pivalate	0.45 ± 0.03											MS-RI	
43.12			unknown 3													
42.93	1460	1460	*(Z)*-methyl isoeugenol					0.15 ± 0.01		0.10 ± 0.01		0.13 ± 0.01			MS-RI	
43.22	1460	1458	alloaromadendrene	3.32 ± 0.21	0.36 ± 0.02		9.82 ± 0.52	0.75 ± 0.05		0.98 ± 0.06				0.15 ± 0.01	MS-RI	
43.33	1489	1490	*β*-selinene									1.05 ± 0.06			Std	
43.51	1492	1492	*γ*-selinene					0.28 ± 0.02						0.31 ± 0.02	Std	
43.55	1498	1499	*α*-selinene	3.81 ± 0.24	0.37 ± 0.02		10.04 ± 0.53	1.11 ± 0.06		1.12 ± 0.08					Std	
43.61	1500	1502	bicyclogermacrene						3.97 ± 0.19	0.14 ± 0.01	1.14 ± 0.07				Std	
43.68	1506	1512	*β*-bisabolene						0.87 ± 0.04						MS-RI	
44.06	1521	1520	dihydroeugenyl butanoate	1.00 ± 0.06	0.88 ± 0.05		0.57 ± 0.04	2.17 ± 1.15		0.24 ± 0.01	1.27 ± 0.08			0.92 ± 0.07	MS-RI	[13]
44.23	1523	1520	*δ*-cadinene				0.92 ± 0.04	1.54 ± 0.07							Std	
44.70	1530	1532	zonarene		0.73 ± 0.04			1.84 ± 0.09	2.60 ± 0.15		1.46 ± 0.09			0.74 ± 0.06	MS-RI	
44.87	1546	1543	*α*-calacorene		0.33 ± 0.02			0.27 ± 0.01		0.28 ± 0.02				0.71 ± 0.05	MS-RI	
44.92	1547	1546	selina-3,7(11)-diene				0.43 ± 0.03	1.29 ± 0.05			0.63 ± 0.05				MS-RI	
45.12	1548	1548	*(Z)*-nerolidol		0.16 ± 0.01			1.85 ± 0.14		0.92 ± 0.07					Std	
45.37	1550	1549	elemol				1.57 ± 0.06	2.11 ± 0.13	5.71 ± 0.28	0.38 ± 0.03	2.16 ± 0.12		3.42 ± 0.18	2.30 ± 0.12	MS-RI	
45.41	1551	1553	ledol							0.33 ± 0.02					MS-RI	
45.89	1578	1579	spathulenol	1.74 ± 0.07	3.40 ± 0.19		0.61 ± 0.05	1.21 ± 0.06	2.86 ± 0.16	7.84 ± 0.36		0.90 ± 0.05		0.29 ± 0.02	MS-RI	
45.97	1580	1581	*Cis*-caryophyllene oxide					0.95 ± 0.05			2.91 ± 0.16	1.11 ± 0.05			MS-RI	
45.97	1583	1583	*Trans*-caryophyllene oxide	5.84 ± 0.26	2.36 ± 0.15		2.55 ± 0.16	9.50 ± 0.41	25.69 ± 1.03	9.20 ± 0.58				1.83 ± 0.10	MS-RI	
46.30	1594	1595	*Cis*-arteannuic alcohol		0.24 ± 0.01										MS-RI	[47]
46.31	1598	1596	carotol		0.22 ± 0.01			0.62 ± 0.05							MS-RI	[44]
46.32	1601	1600	guaiol		0.46 ± 0.03			0.28 ± 0.02		2.03 ± 0.10		0.15 ± 0.01	9.91 ± 0.55		Std	
46.18	1603	1603	*α*-dihydro (10,11)bi sabolol					0.74 ± 0.06		2.14 ± 0.15					MS-RI	
46.48	1606	1608	humulene epoxide II	0.55 ± 0.04	0.97 ± 0.05	5.54 ± 0.22	0.81 ± 0.06	4.68 ± 0.21	4.94 ± 0.08	15.43 ± 0.96	0.62 ± 0.05	0.43 ± 0.03		0.36 ± 0.02	MS-RI	[48]
46.80	1613	1617	isoleptospermone												MS-RI	
46.82	1620	1623	leptospermone		0.26 ± 0.01										MS-RI	[49]
46.92			unknown 4										1.99 ± 0.12			
46.98	1631	1631	dihydroeugenyl pentanoate				0.80 ± 0.05	3.48 ± 0.17	1.05 ± 0.04	0.55 ± 0.04				0.23 ± 0.01	MS-RI	[13]
47.06			unknown 5													
47.15	1632	1634	*γ*-eudesmol		0.47 ± 0.03			2.21 ± 0.13							Std	
47.23			Unknwon 6													
47.32	1641	1643	alloaromadendrene epoxide	0.61 ± 0.05	0.35 ± 0.02			1.68 ± 0.07	1.47 ± 0.06	4.44 ± 0.21		0.05 ± 0.01			MS-RI	
47.42			unknown 7													
47.43	1641	1641	5, *α* caryophylla-4(14),8(15)-dien-5-ol				0.52 ± 0.04	2.19 ± 0.12	2.03 ± 0.13	2.41 ± 0.10		0.15 ± 0.01		0.33 ± 0.02	MS-RI	
47.55	1642	1642	epi-*α*-muurolol					0.83 ± 0.07							MS-RI	
47.83	1644	1644	*α*-selinen-3,11-en-6-ol	0.31 ± 0.02	0.77 ± 0.03		0.65 ± 0.05	3.06 ± 0.19	6.80 ± 0.37	1.80 ± 0.09				0.59 ± 0.04	MS	
47.89	1660	1660	*α*-selinen-11-en-4 -ol	1.18 ± 0.06	0.20 ± 0.01		5.97 ± 0.29	1.53 ± 0.07	12.26 ± 0.62	8.36 ± 0.27		0.81 ± 0.04		0.13 ± 0.01	MS	
48.05	1663	1661	epi-globulol							0.30 ± 0.02		0.03 ± 0.01			MS-RI	
47.97	1675	1670	*β*-bisabolol				0.42 ± 0.03	1.35 ± 0.06	0.95 ± 0.04	0.89 ± 0.04			1.16 ± 0.07		Std	
48.34	1682	1682	ledene oxide II		0.53 ± 0.03			0.33 ± 0.02	0.94 ± 0.05	0.65 ± 0.05				0.13 ± 0.01	MS-RI	[50]
48.56	1700	1713	eudesm-7(11)-en-4-ol		0.24 ± 0.01			0.62 ± 0.05	1.41 ± 0.06	0.22 ± 0.01	0.36 ± 0.02				MS-RI	[51]
49.22			unknown 8													
50.59	1725	1738	*α*-farnesol		1.30 ± 0.06			0.59 ± 0.04	1.59 ± 0.06	0.33 ± 0.02					Std	
51.29	1972	1978	*n*-hexadecanoic acid					0.23 ± 0.01		0.07 ± 0.01					MS-RI	
54.25	2000	1999	eicosane					0.19 ± 0.01		0.85 ± 0.04					MS-RI	
			Number of identified compounds	21	47	8	35	43	20	34	21	39	13	36		

**^z^** ID = Identification methods. MS: by comparison of the Mass spectrum with those of the computer mass libraries Adams, Nist 11 and by interpretation of the mass spectra fragmentations. RI: by comparison of retention index with those reported in literature [8,37]. Std: by comparison of the retention time and mass spectrum of available authentic standards. MS: identification of Mass spectrum. No-polar column ZB-5. Data are the mean of three replicates ± standard deviation. ^y^ Tentatively identified.
